# MicroRNAs as current diagnostic biomarkers and potential therapeutic targets in allergic and non-allergic rhinitis – A functional characterization

**DOI:** 10.1007/s11882-026-01279-0

**Published:** 2026-05-11

**Authors:** Dominik Przychodniak, Wojciech Michał Jankowski, Marcin Kurowski

**Affiliations:** Department of Immunology and Allergy, Medical University of Lodz, Lodz, 90-419 Poland

**Keywords:** MicroRNAs, Allergic Rhinitis, Non-allergic Rhinitis, Airway Inflammation, Nasal mucosa, Epigenetic Regulation

## Abstract

**Purpose of review:**

Allergic rhinitis affects up to one-quarter of the population in industrialized countries. This chronic inflammatory disease of the nasal mucosa is triggered by allergen exposure and mediated by immunoglobulin E, leading to immune dysregulation. Diagnosis typically relies upon skin prick tests, serum-specific immunoglobulin E levels and nasal allergen provocations. Differentiation from non-allergic rhinitis remains a diagnostic challenge. Recent research has identified novel microRNAs, small non-coding RNA regulating post-transcriptional gene expression, as key regulators of immunological pathways with great potential as disease-specific targets in diagnostics and therapy. The purpose of this review is to explore novel therapeutic and diagnostic possibilities regarding novel miRNAs.

**Recent findings:**

This functional review evaluated current evidence specific to miRNA expression in allergic and non-allergic rhinitis. Seven microRNAs (miR-29a, miR-135a, miR-143, miR-146a, miR-150-5p, miR-223, miR-451) are discussed as potential diagnostic markers and therapeutic agents in the future. Interventional studies, including human and animal studies, are reviewed. The available evidence suggested that selected microRNAs may show specificity and sensitivity as biomarkers for allergic and non-allergic rhinitis in future human trials. Therapeutic options involving miRNAs have shown great promise but still lack validation in clinical studies. In this review, we have identified several challenges in microRNA-based diagnostic approaches and suggested strategies to facilitate future development.

**Summary:**

MiRNA research in AR has revealed their role as both diagnostic biomarkers and therapeutic agents in several different mechanisms that are currently under investigation. Dysregulation of miRNA expression has been documented in asthma and allergic rhinitis, but data regarding non-allergic rhinitis remain limited.

## Introduction

Allergic rhinitis (AR) is a chronic, non-infectious inflammatory condition affecting up to 25% of the general population in industrialized countries. AR is typically characterized by inflammation of the nasal mucosa due to allergen exposure, mediated by IgE responses and immune dysregulation. Presence of symptoms in response to allergen exposure, as well as presence of specific IgE, can be ascertained through skin prick testing and/or serum specific IgE assessment. In addition, AR is differentially diagnosed from non-allergic rhinitis (NAR). In these cases, the nasal provocation test (NPT) is used [1–3]. An allergen with suspected culprit is recommended to confirm the diagnosis. However, NAR continues to pose diagnostic and therapeutic challenges. Emerging evidence highlights the pivotal roles of microRNAs (miRNAs) in the pathogenesis, diagnosis, and potential treatment options for AR variants. This review focuses on describing miRNAs implicated in inflammatory and immune processes of AR. We discuss their mechanism of action, diagnostic utility, and therapeutic potential, providing insights into their promise as biomarkers and therapeutic targets for AR in a comparative, methodological revision. Numerous studies demonstrate the importance of miRNAs in regulating inflammatory pathways and their potential as biomarkers. However, little to no is known about the differences in miRNA expression in patients with different types of rhinitis. Therefore, this study review aims to present and evaluate the diagnostic and clinical potential of miRNA expression changes in AR and NAR patients.

**Fig. 1:**
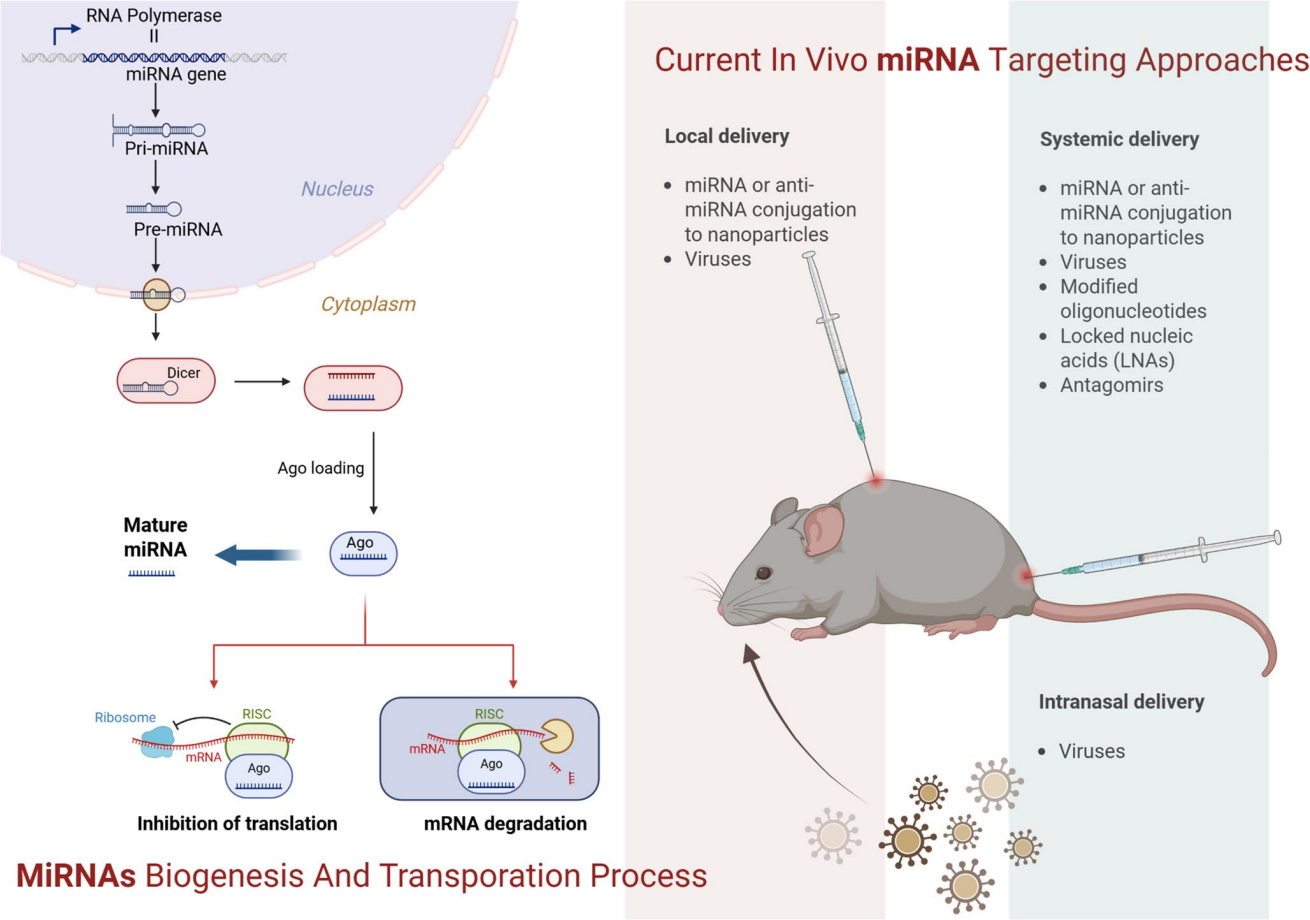
Schematic overview of miRNA biogenesis, intracellular processing, and mechanisms of gene regulation, together with current in vivo miRNA targeting and delivery strategies (local, systemic, and intranasal) illustrated in a mouse model. miRNA genes are transcribed by RNA polymerase II to generate primary miRNA (pri-miRNA), which is cleaved in the nucleus into precursor miRNA (pre-miRNA). The pre-miRNA is exported to the cytoplasm where it is processed into a miRNA duplex. One strand (the guide strand) is incorporated into the Argonaute (Ago)-containing RNA-induced silencing complex (RISC), while the passenger strand is degraded. The mature miRNA–RISC complex binds complementary sequences in target mRNAs, leading to translational repression or mRNA degradation. Current in vivo miRNA targeting approaches are described in greater detail in the text

MicroRNAs (MiRNAs) are small non-coding RNAs (18–22 nucleotides) that play a critical role in post-transcriptional mRNA modulation, thereby regulating gene expression and emerging as critical players in immune and inflammatory pathways, offering potential as diagnostic biomarkers and therapeutic targets (Fig. 1) [4]. miRNAs can regulate the expression of over half of all human genes and are involved in the control of several multi-physiological processes; therefore, dysregulation of miRNA pathways has also been implicated in the pathophysiology of AR. miRNAs regulate key mechanisms of allergic inflammation, including adaptive immune polarization, T cell activation, eosinophil development, and IL-driven epithelial responses [5]. Although they are potential clinical biomarkers, data on their role in rhinitis remain limited, with some studies showing high variability in their expression in bronchial epithelial cells and nasal mucosa across different patient populations with AR phenotypes. This study aimed to identify nasal mucosa biomarkers related to different AR types and assess their potential as surrogate measures of the severity of rhinitis.

## Methodology

A comprehensive literature search was conducted to identify studies evaluating the role of miRNAs as diagnostic biomarkers and therapeutic targets in AR and NAR. Three online databases: PubMed, Scopus, and Google Scholar, were used. A search covered publications between December of 2024 and July of 2025. The database search was conducted using a combination of keywords and Boolean operators. The core search string included the following search terms: (“allergic rhinitis” OR “non-allergic rhinitis”) AND (“microRNA” OR “miRNA”) AND (“biomarker” OR “diagnostic tool” OR “therapeutic target” OR “nasal mucosa”). Additional search terms included “inflammation”, “epithelial barrier”, “immune regulation”, and “airway disease”. Reference lists were also screened to identify additional relevant publications.

Studies were considered eligible if they met the inclusion criteria: original research article or review published in peer-reviewed journals, articles investigating miRNA expression, function, or therapeutic potential in the context of AR, studies involving human subjects, animal models, or in vitro experiments relevant to airway inflammation, and articles published in English. Studies were considered eligible if they did not meet the following exclusion criteria: conference abstracts, editorials, commentaries, or non-peer-reviewed publications; studies irrelevant to miRNA involvement in rhinitis or airway allergic inflammation; articles lacking sufficient methodological description or molecular data on miRNA expression, function, or therapeutic potential.

The titles and abstracts were independently screened by DP and WMJ. Articles not available in full text, meta-analyses, duplicates, and review papers were not further considered. Altogether, 356 studies were identified for further assessment of eligibility. Based on title and abstract information, 55 irrelevant studies, 12 duplicates, 23 reviews, original papers, and meta-analyses were excluded. Finally, this review included 51 publications describing 7 miRNAs. The following information was extracted: miRNA type, involvement of extracellular and intracellular factors, diagnostic and therapeutic potential in the context of rhinitis, and details of the experimental model. Based on the predefined criteria presented above, we have identified 7 miRNAs deemed relevant to the broader context of their potential significance for investigational, diagnostic, and/or therapeutic approaches to allergic rhinitis (Table 1). All authors agreed upon the final selection of sources and miRNAs for further review.

**Fig. 2:**
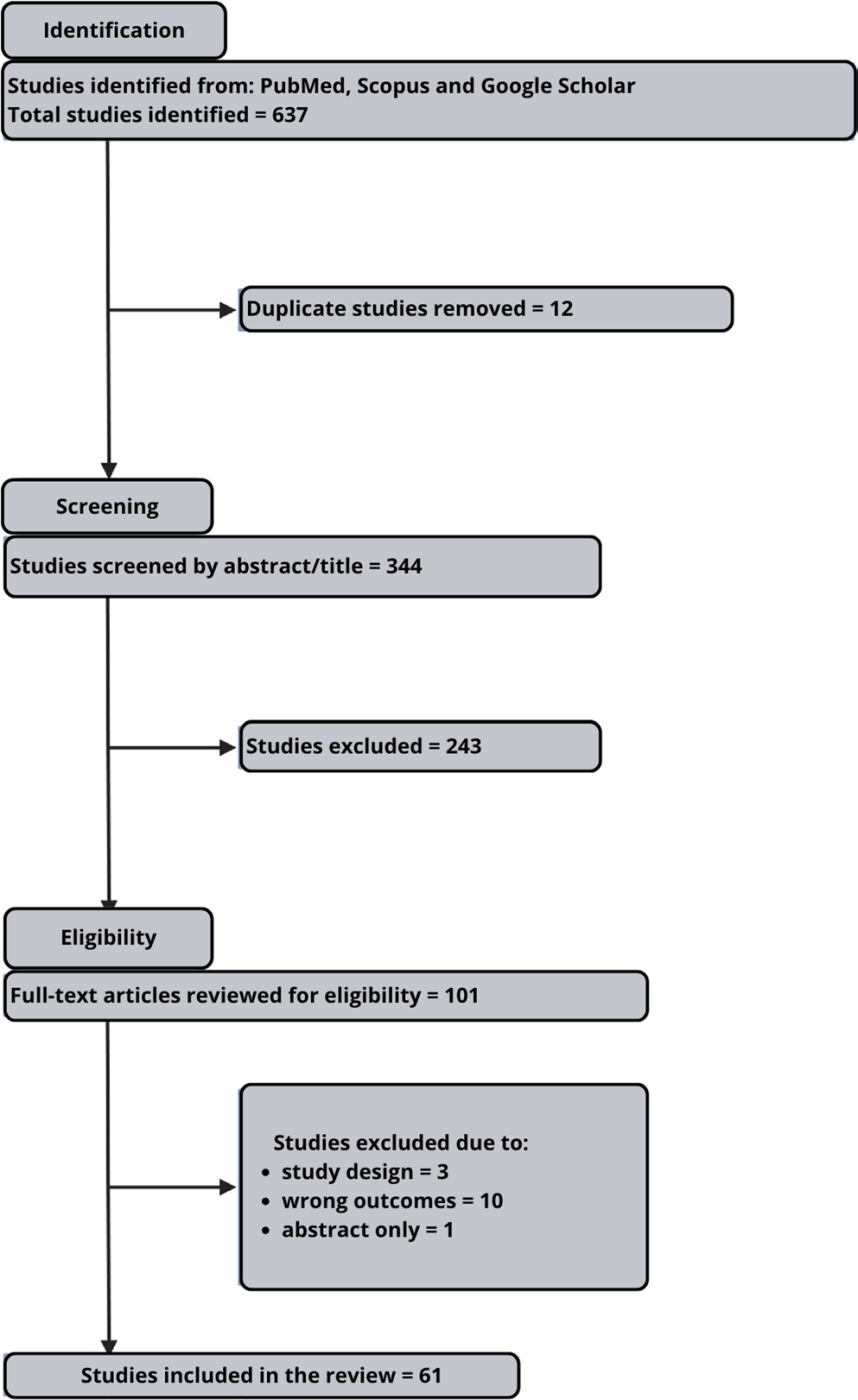
Diagram summarizing the literature search as well as study identification, screening, eligibility, inclusion, and exclusion

Since this review was intended as a narrative review, we did not follow the Preferred Reporting Items for Systematic Reviews and Meta-analyses scheme. The process of source identification and selection based on the above presented criteria and search strings has been summarized in Fig. 2.

**Table 1:** Summary of possible diagnostic and therapeutic potential with respect to the specific mechanism of action of selected microRNAs

miRNA	Involvement of extra- and intracellular factors	Diagnostic potential in AR	Therapeutic potential in AR	Experimental models in studies	Reference
miR-29a	Th2/Th17, IFN-gamma	Upregulated in AR	Antagomir allergen-specific immunotherapy	Human nasal epithelial cells (RPMI2650 and HNEpC)	[6, 7]
miR-135a	Treg/Th17, IL-17, IL-6	Reduced expression linked to AR symptoms worsening	Therapeutic potential not fully confirmed	OVA-induced AR mouse model	g [8–10]
miR-143	IL13Rα1, IL-4	Moderate sensitivity in differentiating NAR	IL13Rα1 suppression by downregulating miR-143	In vitro NECs from AR patients	[11–13]
miR-146a	TNF-alfa, IL-5, IL-13	Lower in asthma and allergy	Insufficient experimental data	Experimental validation in AR models remains limited or unexplored	[14, 15]
miR-150-5p	ICAM-1, IL-10	Expression inversely related to AR severity and ILC2 activation	Nasal administration alleviates AR symptoms via ICAM-1/p38 axis	AR patients and the OVA-induced AR mouse model	[16, 17]
miR-223	ECP, IL-35, NLRP3, IL-18, IL-1beta	Possible neutrophilia and NAR biomarker	Antagomir reduces AR symptoms by restoring INPP4A and reducing inflammation.	Human serum (children and adults with AR), OVA-induced AR mouse model	[18, 19]
miR-451	CCL17, Sirt2	Decreased expression in AR as well as NAR	Experimental data not available	Poly-allergen murine model of allergic airway inflammation	[20, 21]

The primary objective was to identify articles describing the diagnostic and therapeutic potential of miRNAs in allergic and non-allergic rhinitis. Secondary search objectives included identifying articles describing the diagnostic potential of miRNAs with regard to allergic and non-allergic rhinitis. Initially, the titles and abstracts were assessed, and subsequently, the full texts were reviewed. All included studies were published in peer-reviewed English-language journals. 

## Potential Diagnostic Biomarkers

Despite the extensive literature on miRNAs as biomarkers across multiple conditions (e.g., asthma [22–28], chronic obstructive pulmonary disease (COPD) [26, 28–31], cardiovascular diseases [32–35], diabetes [36–40], cancers [41–45], autoimmune diseases [46–48] ), little is known about their potential diagnostic utility in AR [49, 50]. The lack of randomized trials involving the most prominent miRNAs is currently the most important factor limiting the development of novel diagnostic tools. Among reviews published in recent years, most studies have explored the functional and therapeutic roles of MiR-223, MiR-146a, and MiR-150-5p in asthma and allergy [22, 23, 51, 52]. Additionally, single prospective studies have been conducted involving MiR-135a, MiR-143, and MiR-451 in the context of allergic rhinitis. Extensive research on those miRNAs has also been conducted in oncology and hematology. The physiological role of miR-223 is primarily associated with the regulation of macrophage and neutrophil activation and differentiation, as well as the attenuation of excessive inflammatory responses through inhibition of the NF-κB (nuclear factor kappa B) and STAT3 (signal transducer and activator of transcription 3) signaling pathways. miR-223, whose expression is induced by PPARγ (peroxisome proliferator-activated receptor gamma), targets key upstream activators, including IKKα (IkB kinase alpha), STAT3, NLRP3 (NOD-like receptor protein 3), and TRAF6, thereby limiting pro-inflammatory signaling cascades [53]. Through this mechanism, miR-223 suppresses the transcription and secretion of interleukin-6 (IL-6) and tumor necrosis factor-alpha (TNF-α), thereby helping contain immune system hyperactivation. Among the reported targets of miR-223, NLRP3 is a prominent attenuator of cell inflammation, independent of Th2-dependent mechanisms [40–42].

The relationship between miR-223 and immune activity in AR remains unclear. MiR-223 is predominantly expressed in myeloid cells and regulates immune responses by modulating multiple pathways. Abnormal expression of the molecule is shown to be associated with viral hepatitis, tuberculosis, and multiple infectious diseases. In AR, dysregulated miR-223 expression contributes to epithelial barrier dysfunction by impairing tight junction integrity and promoting apoptosis of epithelial cells. This disruption facilitates allergen penetration into the subepithelial tissue, leading to the activation of immune cells and amplification of type 2 inflammatory responses [54]. In the acute phase of inflammation, miR-223 also plays a crucial role in macrophage polarization by promoting the anti-inflammatory M2 phenotype over the pro-inflammatory M1 phenotype. This shift supports the resolution of inflammation and underscores miR-223’s role as a key regulator. Given that changes in miR-223 expression precede macrophage phenotypic switching, it may serve as a more sensitive and earlier biomarker of molecular inflammation, offering potential for timely and targeted therapeutic interventions in AR, as further described in the next paragraph. In a study involving 67 children with moderate-to-severe AR, Ruan et al. demonstrated that serum miR-223 levels were significantly elevated, whereas IL-35 levels were reduced in patients with AR compared with healthy controls. Serum miR-223 levels positively correlated with eosinophil cationic protein (ECP) and peripheral eosinophil counts, while IL-35 levels showed inverse correlations with these markers. An inverse relationship between miR-223 and IL-35 was also observed, with both molecules associated with Th1/Th2 cytokine profiles, eosinophilic inflammation, and clinical severity. In addition to the findings mentioned, the study demonstrated that serum miR-223 levels, measured by real-time PCR, negatively correlated with TNSS (total nasal symptom score) [55]. Given these correlations and the earlier expression changes of miR-223, both miR-223 and IL-35 may serve as promising biomarkers, with miR-223 in particular offering potential as a faster and more sensitive diagnostic tool for AR.

In a 2024 study, Nabil et al. [56]. identified miR-223 as a potential biomarker of AR and of eosinophil-related metabolites. This and other eosinophil-related targets are further addressed in the “Potential Therapeutic Targets” Sect [56]. Another study, conducted by Johnnidis et al. [18], demonstrated that miR-223 plays a pivotal role in regulating neutrophil activation and apoptosis. As mentioned before, neutrophil dysregulation can lead to excessive inflammation and immune suppression. In this study, researchers demonstrated that miR-223 directly targets genes involved in neutrophil differentiation - Mef2c, a transcription factor essential for granulopoiesis. By downregulating Mef2c, miR-223 limited excessive neutrophil production in AR models, preventing neutrophilia [18]. Another important function of miR-223 that might be valuable for early NAR identification is its role in modulating neutrophil activation by targeting NLRP3, a key component of the inflammasome. The NLRP3 inflammasome is responsible for the production of pro-inflammatory cytokines: IL-1β and IL-18. By suppressing NLRP3 expression, miR-223 inhibits excessive inflammasome activation, reducing tissue damage and systemic inflammation during infections and inflammatory diseases [57–61]. MiR-223 regulates neutrophil lifespan by modulating apoptosis; alternatively, dysregulation of miR-223 could be a specific marker of rapid angiogenesis in neoplasms [61]. Circulating miRNAs, including miR-223 and miR-146a, in serum could be developed into panels for systemic biomarker evaluation. MiR-223 has been used in recent studies to evaluate, e.g., plaque instability, colorectal cancer detection, and non-small cell lung disease [62–66]. A study by Langwiński et al. [67] explored miRNA expression in rat models of asthma and AR, providing insights into the regulatory role of miRNAs in airway inflammation. Their findings demonstrated that miR-223-3p expression, a subtype of miR-223 associated with inflammation, is significantly elevated in the lungs of allergic rats exposed to house dust mite allergens. MiR-223-3p, known to target the NF-κB pathway, reduces the expression of inflammatory mediators such as MUC5AC (Mucin 5AC), CCL24 (chemokine c-c motif ligand 24), and TSLP (thymic stromal lymphopoietin), all of which were found to be overexpressed in nasal epithelial cells of AR patients [67]. A study by Zhou et al. [19] confirmed that miR-223-3p expression was significantly elevated in both AR patients and OVA-induced (ovalbumin-induced) AR mice. Consistent with previous findings, intranasal administration of miR-223-3p agomir aggravated allergic symptoms in mice, including increased nasal rubbing and sneezing, OVA-specific IgE, and elevated levels of IL-4, IL-5, and IFN-γ in the nasal mucosa. Histological analysis revealed enhanced eosinophil infiltration. Conversely, administration of the miR-223-3p antagomir alleviated symptoms and reduced inflammation. miR-223-3p was shown to downregulate INPP4A (inositol polyphosphate-4-phosphatase type I A) by directly binding to its 3’UTR.

### MiR-135a and its role in airway inflammation

As part of the miR-135 subgroup (which includes miR-135a and miR-135b), miR-135a is widely known for its involvement in cancer dysregulation mechanisms.^30^ Animal model’s studies of airway inflammation suggest that miR-135a expression alleviate airway inflammation, partially possibly by stimulating the JAK/STAT and STAT3 pathways [68, 69]. A study by Cai et al. has shown that in AR patients, serum miR-135a levels, alongside IL-10, Treg cells, and TGF-beta1, were significantly down-regulated in AR patients compared to healthy individuals. Moreover, patients with elevated serum IgE showed increased levels of Th17 cells, IL-17, and IL-6. Decreased serum miR-135a correlated with Treg/Th17 ratio reduction. Researchers also observed a correlation between lower TNSS scores and elevated serum miR-135a. A serum miR-135a of less or equal to 0.536 was reported to be of diagnostic utility for AR, with a sensitivity of 69.89% and a specificity of 82.89% [8]. Previous AR studies in mice have reported that AR group mice showed nose scratching, sneezing, and watery nasal discharge after OVA challenge, while the control group showed no symptoms. Moreover, AR was induced in mice via intraperitoneal injection and intranasal OVA drops. Luo et al. observed elevated serum OVA-sIgE (ovalbumin-specific immunoglobulin E) concentration in AR mice detected by ELISA 24 h after the last nasal cavity challenge. A study exploring Th1/Th2 imbalance in murine models of AR found that miR-135a levels were higher, whereas IL-4 and GATA-3 levels were lower. The AR group showed higher miR-135a expression levels and correlated with Th1/Th2 imbalance [9].

### Can miRNA be useful in identifying atopic diseases?

The smooth muscle cell (SMC) phenotype is controlled by miRNAs encoded by a bicistronic gene cluster, which includes miR-143 and miR-145 [70]. Previous AR studies in human trials have reported that miR-143 is downregulated in nasal mucosal tissues from AR patients compared with healthy control subjects [11]. Teng et al. reported miR-143 repressed secretion of inflammatory cytokines and mucus in IL-13-stimulated NECs (nasal epithelial cells) from AR patients by targeting IL13Rα1. They obtained nasal mucosa samples from 23 patients with AR and 18 patients with NAR. In this study, induced expression of miR-143 significantly decreased mRNA and protein expression levels. Inhibited expression of GM-CSF (granulocyte-macrophage colony-stimulating factor), eotaxin, and MUC5AC (mucin 5AC) in IL-13-stimulated NECs was observed. In discriminating AR from NAR, miR-143 showed moderate accuracy, with a sensitivity of 82.6% and a specificity of 69.6%. Moreover, miR-143 directly targeted and significantly suppressed IL-13 receptor α1 chain, thereby correcting the Th1/Th2 imbalance [13]. MiR-143 as a potential target for the prevention and treatment of AR will be further described in the Potential Therapeutic Targets section.

MiR-146a acts as a negative regulator of inflammation by targeting TRAF6 (Tumor necrosis factor receptor associated factor 6) and IRAK1 (interleukin 1 receptor associated kinase 1), intermediates in the TLR (Toll-like receptor) and cytokine receptor pathways [71]. By modulating these pathways, miR-146a suppresses the activation of NF-κB and the production of inflammatory cytokines. By binding to the 3’ untranslated regions of IRAK1 and TRAF6 mRNA, miR-146a suppresses their translation, attenuating the activation of NF-κB. This suppression results in decreased production of pro-inflammatory cytokines, including IL-1β, IL-6, and TNF-α [14, 72]. The feedback inhibition by miR-146a prevents excessive inflammatory responses, a mechanism that is particularly relevant in AR, where inflammation plays a central role. Luo et al. found that miR-146a levels in nasal lavage fluids from AR patients correlated with eosinophilic inflammation and Th2 cytokine activity, including elevations in IL-5 and IL-13. The study highlighted the potential of miR-146a analysis in nasal fluids as a biomarker for AR severity and disease progression. Nasal lavage sampling provides a localized and minimally invasive diagnostic approach. MiR-146a’s dynamic response to allergen exposure further enhances its diagnostic value [73].

Wardzyńska et al. have assessed circulating miRNA expression in asthmatic patients across various age groups (including elderly subjects) and its association with clinical, inflammatory, and respiratory parameters, without, however, making provision for co-existent rhinitis. Researchers have demonstrated lower expression of miR-146a, -126a, -106a and − 19b in elderly patients with well documented asthma. MiR-146a expression was significantly lower in elderly patients with asthma, compared to healthy individuals and non-elderly asthmatics. Interestingly, serum miRNA levels were found to correlate with asthma clinical symptoms’ intensity, their control level as well as other systemic inflammatory indices (e.g., TNF-alpha) in elderly asthmatic subjects [15].

### miR-150-5p as the ICAM-1/p38 axis inhibitor

The function of miR-150-5p in the pathogenesis of AR appears to be closely linked to the regulation of type 2 innate lymphoid cells (ILC2s). Studies in a mouse model of AR suggest multiple possible pathways for the interaction between miR-150-5p and ILC2s, namely through ICAM-1, GATA-3, and up-regulation of specific IgE (sIgE). A study involving an AR mouse model and AR patients was conducted by Zhang et al. The AR mouse model was established using the OVA challenge, and miR-150-5p, ICAM-1 (intercellular adhesion molecule-1), p-p38 (phosphorylated p38 MAPK), and p-GATA-3 (phosphorylated GATA-binding protein 3) expressions were assessed, the ILC2s levels were measured, and TNSS was collected from all participants in two stages of the study - lentiviral miR-150-5p administration and lentivirus-ICAM-1 administration. Decreased miR-150-5p expression and increased ICAM-1, p-p38, p-GATA-3, and ILC2s levels were observed compared to controls [16]. Results showed that treatment with a miR-150-5p lentivirus alleviated AR symptoms, reduced mucosal inflammation, and lowered ILC2 numbers. The study explored molecular and subjective changes in 40 AR patients and 20 AR mice following nasal treatment with miR-150-5p [16, 74]. This therapeutic approach will be discussed in greater detail in the next paragraph. Although a study by Neamah et al. addressed the correlation between myeloid-derived suppressor cells (MDSCs) and the downregulation of miR-150-5p and miR-543-3p, it demonstrated that these two miRNAs targeted and enhanced anti-inflammatory genes, including IL-10, PIM1, ARG2, and STAT3, broadening the molecular scope of potential targeted therapy for miR-150-5p [17]. Documented negative correlation between ILC2-related pathway activation and miR-150-5p expression suggests further research.

### Overexpression of miR-29a in AR

The miR-29 family, consisting of miR-29a, miR-29b, and miR-29c, has been associated with malignant neoplasms and may serve as a biomarker for predicting the aggressiveness of cancers [75–78]. Yet, the function of miR-29a in non-malignant diseases had not been sufficiently summarized. In a study identifying differentially expressed miRNAs correlated with mRNA levels of candidate inflammatory genes, Wei et al. have shown a negative correlation between miR-29a and PTEN (phosphatase and tensin homolog), a critical regulator of inflammatory and cellular signaling pathways. Downregulated PTEN promotes a proinflammatory state and correlates with elevated cytokine levels.^63^ Further studies provided novel insights, demonstrating that miR-29a may play a role in AR pathology in yet another mechanism. FOS is a phosphoprotein produced by c-fos transcription and plays an important role in cell proliferation and the apoptosis cycle. MiR-29a was found to regulate the proliferation of the human nasal epithelial cell line through post-transcriptional modulation of FOS [6]. Fan et al. observed that miR-29a expression was upregulated, whereas FOS mRNA expression was downregulated, in nasal tissues from AR patients compared with healthy controls. miR-29a overexpression was associated with increased proliferation and reduced apoptosis in in vitro cell lines [79]. Interestingly, miR-29 appears to mediate innate and adaptive immune responses to bacterial and viral infections, for example, by modulating IFN-gamma production, although the mechanism remains incompletely understood [80]. Specjalski et al. assessed expression of 740 miRNAs before and 24 h after the first allergen immunotherapy with wasp venom in 7 patients. Expression of 5 miRNAs changed significantly, and among them was miR-29c [81]. Results suggest that higher expression of miR-29c, known to be involved in allergic inflammation, could contribute to tolerance induction, creating space for potential miRNA therapeutic approaches, described later in this review. As mentioned before, miR-29a regulates FOS expression, and both miR-29a overexpression and FOS silencing promoted cell proliferation and inhibited apoptosis. Co-transfection with viral vectors of miR-29a and FOS reduced proliferation and increased apoptosis. These findings suggest that miR-29a may influence nasal epithelial cell behavior in AR. Another study by Wang et al. investigated potential target genes of miR-29a-3p and the effects of its suppression in the OVA-induced AR mouse model. Two proteins most strongly associated with adherent junctions (AJs) and tight junctions (TJs) of nasal mucosal epithelial cells were VCL and CTNNB1 [82].

### Allergic and non-allergic expression of miR-451

MiR-451 is a distinctive microRNA that undergoes an Ago2-dependent cytoplasmic biogenesis pathway and has been reported to participate in cancer-related pathways and other pathological processes, including arthritis and chronic obstructive pulmonary disease (COPD) [7, 83–85]. A study by Adamczyk et al. analyzed the expression of miR-146a and miR-16 in the nasal mucosa of 16 allergic and 21 non-allergic children with adenoid hypertrophy (AH) who underwent tonsillectomy. It was observed that miR-146a expression in the adenoid mucosa of allergic patients was significantly decreased compared with non-allergic patients (*p* = 0.019) [20]. This differential expression suggests a unique regulatory mechanism linking miR-451 dysregulation to AR rather than NAR. Studies by Chung et al. provided novel insights, demonstrating that oxidant stress increases miR-451 expression in macrophages while reducing Ago2 protein levels. They highlighted that, while the biological functions of miR-451 are increasingly understood, its role in allergic diseases remains unexplored [86]. Further studies by Chung et al. revealed that miR-451 plays a major role in regulating macrophage phenotype in allergic airway inflammation. Researchers using an experimental polyallergenic murine model found that miR-451 contributed to the allergic induction of CCL17 (C-C motif chemokine ligand 17) in the lung and promoted pro-asthmatic macrophage activation. Additionally, administration of a Sirt2 (Sirtuin 2) inhibitor reduced alternate macrophage activation and significantly suppressed lung inflammation induced by triple allergens (dust mite, ragweed, and Aspergillus fumigatus) [21, 86]. These findings suggest that miR-451, by influencing allergen-mediated macrophage phenotypes, could serve as a potential diagnostic target for AR and NAR in the future.

## Potential Therapeutic Targets

In this section, we will further describe the aforementioned therapeutic potential of selected miRNAs and provide novel insights into recent interventional studies of miRNA utility in AR and NAR in animal models and/or human studies. Basic science and mechanisms of the mentioned miRNAs have already been described in the previous paragraph. The literature on potential therapeutic targets of miRNAs remains insufficient. This paragraph aims to point out the starting point for further research. 

### Perspectives for miR-223-3p inhibitors implementation

In a study using a PCLS (precision-cut lung slice) model of asthma, Nowakowska et al. investigated the effects of inhibition of miR-223-3p and miR-328a-3p on allergic inflammation. PCLSs were generated from rats with HDM-induced allergic inflammation and from controls, then transfected with miRNA inhibitors. The study showed significantly decreased expression of IL-33, Ccl5 (C-C motif chemokine ligand 5), Prg2, and TSLP in both allergic and healthy rats compared with non-transfected controls. At the same time, no significant differences in protein levels were detected in the culture medium [87]. These novel findings show that not only do inhibitors of miR-223 modulate inflammation in airway allergic diseases, but their mechanism of action is also independent of allergic stimulation. In a study involving 67 children with AR, Ruan et al. observed elevated serum miR-223 levels and reduced IL-35 levels in AR patients, with both markers strongly associated with inflammatory indicators and clinical severity. Authors suggest that modulating miR-223 expression or restoring IL-35 levels could offer help to correct immune imbalance and reduce symptom burden in AR, as described in the previous paragraph [55]. Several studies have demonstrated that miR-223 mimics can effectively reduce inflammation in preclinical models of acute lung injury (ALI) and viral myocarditis [53, 54]. MiR-223’s ability to modulate macrophage polarization, favoring the anti-inflammatory M2 phenotype over the pro-inflammatory M1 phenotype, was also described by Qu et al. in a series of rat model studies [55, 88–90]. Qu et al. observed that EAU (experimental autoimmune uveitis) rats treated with miR-223-3p or the Notch signaling inhibitor DAPT (a γ-secretase inhibitor) exhibited similar inhibitory effects on the pathological process. Both treatments suppressed Notch pathway activation by modulating RBPJ (recombination signal-binding protein Jκ), thereby restoring the M1/M2 macrophage polarization balance. MiR-223 contributed to tissue protection by maintaining epithelial barrier integrity. In pulmonary fibrosis models, miR-223 was shown to reduce epithelial damage and fibrosis progression by regulating pathways involved in cellular apoptosis and oxidative stress. Although specific data on miR-223’s therapeutic application in AR are limited, its demonstrated efficacy in related inflammatory diseases provides a strong rationale for exploring its use in AR. In studies by other researchers, targeted delivery systems, such as nanoparticles and liposomes, are being developed to enhance the precision and efficacy of miR-223-based therapies [91]. In a mouse model of AR, Zhou et al. demonstrated that miR-223-3p directly contributes to disease progression by targeting INPP4A. Intranasal administration of miR-223-3p agomir intensified allergic responses, increasing OVA-specific IgE, nasal inflammation, and type 2 cytokine levels. As suspected, treatment with a miR-223-3p antagomir significantly reduced allergic symptoms, inflammatory markers, and eosinophil infiltration in the nasal mucosa [19].

### Intranasal miR-135a administration

Deng et al. conducted a study with intranasal, lentiviral miR-135a in an AR mouse model. Expression of GATA-3 was lower in the AR mice model following miRNA treatment. Notably, an increase in the percentage of Th1 cells and a decrease in the percentage of Th2 cells were also observed. Results suggest that miR-135a treatment not only significantly modulated protein expression but also appeared to balance the Th1/Th2 ratio in the AR mouse model [10]. Previous investigations have shown that mast cell suppression in mice treated with intranasal miR-135a was also caused by downregulation of GATA-3 expression in allergen-induced inflammation [9]. It seems that miR-135a might be readily used in intranasal therapy to treat the allergic phenotype of AR. Additional work exploring the full therapeutic potential of miR-135a in the treatment of allergen-induced inflammation is suggested.

### miR-143

As mentioned in the previous paragraph regarding miR-143, forced expression of miR-143 inhibited mRNA and protein expression of GM-CSF, eotaxin, and MUC5AC. Furthermore, because miR-143 expression is more significantly decreased in AR patients than in NAR patients, it’s suggested that a potential miR-143 therapeutic intervention would be more beneficial in AR patients. In an in vitro study conducted by Yu et al., miR-143, along with miR-106b, was found to regulate dendritic cells in a pro-allergic state negatively, thus alleviating AR symptoms. Directly, downregulation of miR-143 in AR was found to suppress the IL-13 receptor alpha-1 chain gene [12]. Therefore, reduced IL-13 expression in nasal epithelial cells could be a potential therapeutic mechanism, with miR-143 as a candidate for AR treatment.

### Evidence for miR-29a-3p promotion of nasal epithelial barrier dysfunction

As described in the previous paragraph, results from Wang et al. have contributed to the identification of a potential novel therapeutic target for the treatment of AR. Researchers found that in OVA-induced AR mouse models, inhibition of miR-29a-3p and, subsequently, its target genes VCL and CTNNB1, resulted in partial alleviation of AR symptoms. MiR-29a-3p antagomir could potentially restore mucosal integrity and barrier function. Wang et al. compared the effects of intranasal administration of miR-29 agomir and mismatched intranasal treatment of agomir (miR-MA) on AR mice. Compared to the control group, nasal mucosal tissues after miR-29 overexpression showed a significant decrease in nasal friction and sneezing. Moreover, histopathological changes in AR mice following miR-29 overexpression were investigated. Researchers reported that restoring miR-29 significantly reduced eosinophil infiltration and apoptosis in the nasal mucosa. Not only was restoration associated with suppression of epithelial apoptosis, but mRNA expression of relevant inflammatory factors, including IL-6, Cleaved caspase-3, IL-10, and IFN-gamma, was also found to be decreased upon miR-29 agomir treatment [7]. In another study, Specjalski et al. suggested miR29-c as a potential target for novel therapies in wasp venom immunotherapy (VIT) after results from a pilot study analyzing 740 miRNAs in 7 adult patients with a history of severe systemic reactions after wasp stings. Significant changes in miR-29c expression have been found as a result of the buildup phase of wasp VIT. Results suggest that higher expression of miR-29c, known to be involved in allergic inflammation, could contribute to tolerance induction, creating space for potential miRNA therapeutic approaches. Although the role of the most significant changes remains uncertain, researchers highlight allergen-specific immunotherapy as the most effective treatment as of today [81]. It’s speculated that, in the future, immunotherapy could be conducted using safer, more effective miRNAs, with miR-29a as a potential candidate.

### miR-150-5p as the ICAM-1/p38 axis inhibitor

Zhang et al. conducted a study involving 40 patients with AR and 20 AR model mice that were treated with nasal administration of miR-150-5p lentiviruses and negative control (NC) lentiviruses. Treatment in both groups led to alleviation of AR symptoms. ILC2s function suppression, downregulation of ICAM-1 expression, and significantly elevated levels of miR-150-5p were observed in AR patients (*p* < 0.001 vs. control). In the next phase of the study, which involved only an animal model of AR, the lentiviral ICAM-1 was administered, resulting in reversal of the molecular changes (described in the previous paragraph) and alleviation of symptoms [16].

## Future Directions

**Fig. 3:**
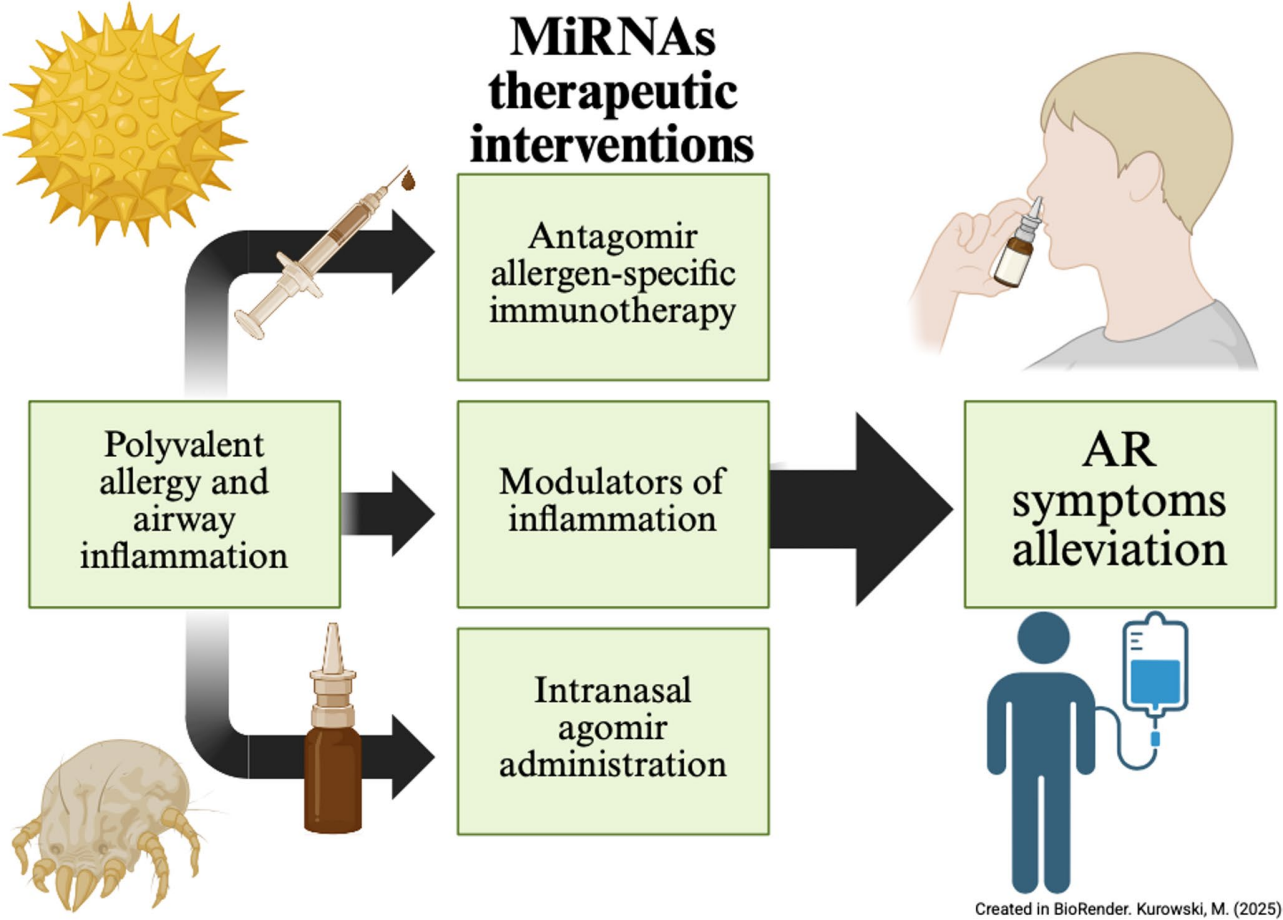
Schematic representation of potential applications and therapeutic strategies of miRNA-based approaches in AR. MiRNA research in AR has revealed their role as both diagnostic biomarkers and therapeutic agents across several mechanisms currently under investigation. From one perspective, antagomir allergen-specific immunotherapy has been proposed as an enhanced desensitization therapy. It could contribute to improved treatment responses or even serve as a sole treatment option in the future, although clinical studies are still lacking at the time of this review. On the other hand, alleviation of AR symptoms through miRNA modulators, which are found to be downregulated in children with AR and have shown promising results in asthma, could be useful for symptom-targeted interventions in AR. As of now, delivery systems and safety issues need to be resolved to realize the promise of miRNA-based therapies fully. When it comes to intranasal agomir administration, research represents a novel approach for restoring deficient miRNA activity. For lentiviral vectors, safe delivery systems remain in preclinical testing. The central function of miRNAs in AR centers on inflammatory modulation, particularly their role as key regulators of neutrophilic and eosinophilic activity. Finally, poly-allergen airway inflammation could be addressed by miRNAs, which have already demonstrated clinical utility in detecting NAR with high sensitivity and specificity, as described in the previous paragraph of this review

Advances in miRNA-based diagnostics and therapeutics hold great promise for long-term AR management. However, challenges, including optimizing delivery systems, ensuring specificity, and minimizing off-target effects, remain to be overcome. Future research should focus on longitudinal studies to validate miRNA biomarkers, standardize miRNA-level tests, and conduct clinical trials to establish the safety and efficacy of miRNA-based therapies. Lower expression of miR-451 has been assessed in AH children with AR, yet no studies addressing potential therapeutic utilities have been conducted. Similarly, miR-146a has been shown in asthma to be a reliable biological biomarker but lacks experimental validation in AR models. Furthermore, no data on the therapeutic potential of miR-146a are available, and research should begin by assessing the safety of potential miR-146a agomirs. On the other hand, miR-223 has been extensively studied for its diagnostic and therapeutic utility, with many potential applications identified, such as serving as a neutrophilic biomarker and a molecular differentiator of NAR. Although the pathway from antagomir use in OVA-induced AR mice to a commercially available and effective drug is still far from the end, miR-223-3p inhibitors are recognized in this review as the most promising miRNA in the context of NAR research. Also, regarding miR-150-5p, nasal lentiviral administration shows great potential for future therapeutic approaches but requires clinical studies with a larger number of groups. MiR-143 was the only one of the mentioned miRNAs that was proven in clinical trials to be sensitive and specific for the detection of NAR; thus, more research on its diagnostic utility should be initiated. MiR-29a antagomir allergen-specific immunotherapy, as well as diagnostic potential in AR, could be beneficial, and trials regarding the effectiveness of such a biomarker or immunotherapeutic agent are needed (Fig. 3). At the time of writing this review, no miRNA-based immunotherapies other than those described in the context of VIT have been reported in the literature.

Together, these findings underscore the potential of miRNA-based therapeutic and diagnostic options for future AR management. However, directly translating agomir and antagomir approaches from experimental models into clinical applications would require lengthy, costly optimization research trials; the potential benefits most certainly outweigh the costs associated with the process.

Finally, four main obstacles standing in the way of the development of miRNA-based diagnostic and therapeutic approaches have been identified:

- Lack of optimized delivery systems, including lentiviral vectors.
- Persistent specificity of action in terms of miRNA regulation.
- Standardized methods for miRNA quantification.
- Lack of longitudinal and clinical trials.

## Key References

- Langwiński W, Szczepankiewicz D, Narożna B, Stegmayr J, Wagner D, Alsafadi H, Lindstedt S, Stachowiak Z, Nowakowska J, Skrzypski M, Szczepankiewicz A. Allergic inflammation in lungs and nasal epithelium of rat model is regulated by tissue-specific miRNA expression. Mol Immunol. 2022 Jul;147:115-125. doi:10.1016/j.molimm.2022.04.01 .
  - ○ This experimental study demonstrates tissue-specific miRNA expression patterns in allergic airway inflammation, thus supporting the concept of localized miRNA biomarkers in nasal mucosa and lower airway tissues that are sensitive and specific for diagnostic approaches.
- Li K, Jin J, Yang Y, Luo X, Wang Y, Xu A, Hao K, Wang Z. Application of Nanoparticles for Immunotherapy of Allergic Rhinitis. Int J Nanomedicine. 2024 Nov 18;19:12015-12037. doi: 10.2147/IJN.S484327.
  - ○ This review summarizes advances in nanoparticle-based immunotherapy and drug delivery systems, which are highly relevant for the clinical translation of miRNA-based therapeutics in allergic rhinitis.
- Zhang L, Meng W, Chen X, Ning Y, Sun M, Wang R. MiR-150-5p regulates the functions of type 2 innate lymphoid cells via the ICAM-1/p38 MAPK axis in allergic rhinitis. Mol Cell Biochem. 2022 Apr;477(4):1009-1022. doi: 10.1007/s11010-021-04346-4.
  - ○ This study provides experimental evidence that miR-150-5p modulates ILC2-driven inflammation through the ICAM-1/p38 pathway, supporting its role as both a biomarker and therapeutic target in allergic rhinitis.

## Data Availability

No datasets were generated or analysed during the current study.
